# A DEAD-box RNA helicase mediates meiotic silencing by unpaired DNA

**DOI:** 10.1093/g3journal/jkad083

**Published:** 2023-04-13

**Authors:** Victor T Sy, Erin C Boone, Hua Xiao, Michael M Vierling, Shannon F Schmitz, Quiny Ung, Sterling S Trawick, Thomas M Hammond, Patrick K T Shiu

**Affiliations:** Division of Biological Sciences, University of Missouri, Columbia, MO 65211, USA; Division of Biological Sciences, University of Missouri, Columbia, MO 65211, USA; Division of Biological Sciences, University of Missouri, Columbia, MO 65211, USA; Division of Biological Sciences, University of Missouri, Columbia, MO 65211, USA; Division of Biological Sciences, University of Missouri, Columbia, MO 65211, USA; Division of Biological Sciences, University of Missouri, Columbia, MO 65211, USA; Division of Biological Sciences, University of Missouri, Columbia, MO 65211, USA; School of Biological Sciences, Illinois State University, Normal, IL 61790, USA; Division of Biological Sciences, University of Missouri, Columbia, MO 65211, USA

**Keywords:** DEAD-box RNA helicase, genome defense, meiotic silencing by unpaired DNA (MSUD), *Neurospora crassa*, RNA interference (RNAi)

## Abstract

During the sexual phase of *Neurospora crassa*, unpaired genes are subject to a silencing mechanism known as meiotic silencing by unpaired DNA (MSUD). MSUD targets the transcripts of an unpaired gene and utilizes typical RNA interference factors for its process. Using a reverse genetic screen, we have identified a meiotic silencing gene called *sad-9*, which encodes a DEAD-box RNA helicase. While not essential for vegetative growth, SAD-9 plays a crucial role in both sexual development and MSUD. Our results suggest that SAD-9, with the help of the SAD-2 scaffold protein, recruits the SMS-2 Argonaute to the perinuclear region, the center of MSUD activity.

## Introduction

The filamentous fungus *Neurospora crassa* is primarily haploid, with a transient diploid sexual stage (Shear and Dodge 1927; McClintock 1945; Raju 2009). It grows as an intertwined, coenocytic network of tube-like cells called hyphae (Fischer and Glass 2019). While this vegetative lifestyle may be beneficial for resource distribution, selfish genetic elements can potentially spread throughout the colony. As a defense strategy, *Neurospora* has evolved several genome surveillance mechanisms (Gladyshev 2017), including one called meiotic silencing by unpaired DNA (MSUD) (Shiu *et al*. 2001; Hammond 2017). In this mechanism, a gene not having a meiotic pairing partner is perceived as a potential intruder and is silenced during sexual development.

MSUD begins at meiotic prophase I, when homologous chromosomes engage in a direct dsDNA–dsDNA (double-stranded DNA) pairing process that may involve SAD-6 (chromatin remodeler) and REC8 (meiotic kleisin) (Samarajeewa *et al*. 2014; Rhoades *et al*. 2021). When an unpaired region is detected, a single-stranded aberrant RNA (aRNA) is transcribed and exported to the perinuclear region, where it is converted into a double-stranded RNA (dsRNA) by SAD-1 (RNA-directed RNA polymerase) (Shiu and Metzenberg 2002). SAD-3, an RNA helicase, may help SAD-1 to clear secondary structures of the aRNA template (Hammond, Xiao, Boone, *et al*. 2011). The dsRNA is cleaved by DCL-1 (Dicer) into small interfering RNAs (siRNAs), which are turned into single strands by QIP (exonuclease) (Alexander *et al*. 2008; Xiao *et al*. 2010; Hammond, Spollen, *et al*. 2013). The single-stranded siRNAs then guide SMS-2 (Argonaute) to complementary mRNAs, in a process that appears to be mediated by nuclear cap-binding proteins NCBP1/2/3 (Lee *et al*. 2003; Decker *et al*. 2017; Boone *et al*. 2020). SAD-2 (scaffold protein) functions to tether SAD-1 and others to the perinuclear region (Shiu *et al*. 2006; Decker *et al*. 2015). CAR-1 and CGH-1, two RNA granule proteins, are also involved in meiotic silencing (Xiao *et al*. 2021).

DEAD-box RNA helicases contain the Asp-Glu-Ala-Asp (DEAD) motif and depend on ATP for their RNA-binding ability (Linder and Fuller-Pace 2015; Valentini and Linder 2021; Weis and Hondele 2022; Naineni *et al*. 2023). They can unwind short duplex RNAs, disrupt RNA–protein interactions, and/or act as platforms for protein assembly. Members of this group of proteins include Belle and Vasa, which are components of perinuclear nuage granules in *Drosophila melanogaster* germ cells (Gao and Arkov 2013; Lasko 2013) and have been implicated in RNA silencing (Dehghani and Lasko 2017). In this work, we have identified an MSUD protein with similarity to Belle and Vasa and explored its role in silencing.

## Materials and methods

### Fungal manipulation and genotypes

The Neurospora online protocol guide (https://www.fgsc.net//neurospora/neurosporaprotocolguide.htm) was followed during the course of this study. Genotypes of *Neurospora* strains used are listed in Table 1. Various progenitor strains, including those from the knockout library (Dunlap *et al*. 2007), were obtained from the Fungal Genetics Stock Center (FGSC) (McCluskey *et al*. 2010). Growth and crossing media were prepared using established protocols (Westergaard and Mitchell 1947; Vogel 1956). Linear growth rates were measured at room temperature with race tubes (Turner 2011). Quantification of ascospore (sexual spore) production was conducted according to Hammond, Xiao, Boone, *et al*. (2011).

**Table 1.** *Neurospora* strains used in this study.

Strain	Genotype
F2-01	*fl A* (FGSC 4317)
F2-29	*rid r* ^Δ^ *::hph; fl A*
F5-06	*fl; gfp-sms-2::hph a*
F9-03	*sad-9* ^Δ^ *::hph; fl A*
P3-08	Oak Ridge wild type *a* (FGSC 2490)
P15-14	*rid his-3; mus-52* ^Δ^ *::bar; gfp-sms-2::hph A*
P22-30	*mCherry-sad-9::nat1 rid; mus51* ^Δ^ *::bar A*
P22-31	*mCherry-sad-9::nat1 rid his-3; mus51* ^Δ^ *::bar a*
P22-36	*mCherry-sad-9::nat1 rid; mus51* ^Δ^ *::bar*; *sad-2*^Δ^*::hph A*
P22-37	*mCherry-sad-9::nat1 rid; mus51* ^Δ^ *::bar*; *sad-2*^Δ^*::hph a*
P22-38	*mCherry-sad-9::nat1 rid; sad-2-gfp::hph a*
P22-39	*mCherry-sad-9::nat1 rid; sad-2-gfp::hph A*
P22-40	*mCherry-sad-9::nat1 rid nup120-gfp::hph; mus51* ^Δ^ *::bar a*
P22-41	*mCherry-sad-9::nat1 rid nup120-gfp::hph his-3; mus51* ^Δ^ *::bar A*
P22-47	*mCherry-sad-9::nat1 rid; mus51* ^Δ^ *::bar; gfp-sms-2::hph a*
P22-48	*mCherry-sad-9::nat1 rid; gfp-sms-2::hph A*
P22-49	*sad-9* ^Δ^ *::hph r* ^Δ^ *::hph a*
P23-09	*sad-9* ^Δ^ *::hph a*
P27-22	*sad-9* ^Δ^ *::hph; gfp-sms-2::hph A*
P27-23	*sad-9* ^Δ^ *::hph; gfp-sms-2::hph a*
P27-24	*sad-9* ^Δ^ *::hph; sad-2-gfp::hph A*
P27-25	*sad-9* ^Δ^ *::hph; sad-2-gfp::hph a*
P27-32	*yfpc-sad-9::hph rid; yfpn-sms-2::hph A*
P27-33	*yfpc-sad-9::hph rid; yfpn-sms-2::hph a*
P27-36	*yfpc-sad-9::hph rid; yfpn-sad-2::hph A*
P27-37	*ypfc-sad-9::hph rid his-3; yfpn-sad-2::hph a*
P27-38	*sad-9* ^Δ^ *::hph a* (FGSC 18466)
P28-47	*rid; sad-2-gfp::hph a*
P28-48	*rid; sad-2-gfp::hph A*

Genetic loci are described in the *N. crassa* e-Compendium (http://www.bioinformatics.leeds.ac.uk/∼gen6ar/newgenelist/genes/gene_list.htm).

### Reverse genetic screen and MSUD assay

High-throughput screening of the *Neurospora* knockout library for MSUD mutants and the subsequent quantitative analysis of silencing suppression were as previously reported (Hammond, Xiao, Boone, *et al*. 2011; Xiao *et al*. 2019). The aforementioned screening involved the mating of knockout strains with an *asm-1*^Δ^ tester and an *r*^Δ^ tester, with the latter identifying the MSUD-deficient mutant described in the *Results* section.

### Sequence analysis

The sequence, gene model, and location of *sad-9* (*ncu09093-t26_1*) can be found in FungiDB (Basenko *et al*. 2018). With the online BLASTP v2.13.0+ software (basic local alignment search tool for proteins; Camacho *et al*. 2009), the SAD-9 amino-acid sequence was used to search the National Center for Biotechnology Information's model organisms database for homologs and functional domains.

### Expression analysis

To compare the transcript levels of different MSUD genes, *Neurospora* vegetative (SRR080688, SRR081479, SRR081546, and SRR081586) and sexual (SRR957218) RNA-seq datasets from Ellison *et al*. (2011) and Samarajeewa *et al*. (2014) were downloaded and analyzed, following a published procedure (Decker *et al*. 2017).

### Strain construction and confirmation

Standard molecular techniques were used throughout this investigation (Sambrook and Russell 2001). Green fluorescent protein (GFP) and mCherry tagging constructs were created using double-joint polymerase chain reaction (DJ-PCR) (Hammond, Xiao, Rehard, *et al*. 2011; Samarajeewa *et al*. 2014). *Neurospora* transformation by electroporation of conidia (asexual spores) was adapted from Margolin *et al*. (1997). For strain confirmation, fungal DNA was extracted from conidia (Henderson *et al*. 2005) or hyphae (Qiagen DNeasy Plant Mini Kit). For PCR-based genotype screening and verification, the Promega GoTaq Green Master Mix or the Roche Expand Long Range dNTPack was used. DNA sequencing was carried out by the University of Missouri (MU) Genomics Technology Core. Primers for this study are listed in Supplementary Table S1.

### Bimolecular fluorescence complementation

As an *in vivo* protein–protein interaction assay, bimolecular fluorescence complementation (BiFC) relies on the reconstitution of the yellow fluorescent protein (YFP) when its nonfluorescing halves are brought together by two interacting proteins (Hu *et al*. 2002; Bardiya *et al*. 2008). YFP halves were tagged in the manner of Hammond, Xiao, Rehard, *et al*. (2011).

### Photography and microscopy

Z-stack pictures of protoperithecia (female structures) were obtained from an M205 FA stereomicroscope and a DFC345 FX camera from Leica. For photography of perithecia (fruiting bodies), an Apple iPhone 5 with a Magnifi photoadapter (Arcturus Labs, Lawrence, KS, USA) and a Vanguard 1231CM microscope were utilized. Images of asci (spore sacs) were captured using an Olympus DP74 camera attached (via a 0.63× C-mount) to an Olympus BX45 microscope. For fluorescence microscopy, preparation and visualization of asci were performed as previously described (Alexander *et al*. 2008; Xiao *et al*. 2010), with the employment of a Leica TCS SP8 system at the MU Advanced Light Microscopy Core.

## Results

### *ncu09093* ^Δ^ is a semidominant MSUD suppressor

To expedite research in the MSUD pathway, we have developed a high-throughput reverse genetic screen to identify silencing mutants from the *Neurospora* knockout library (Hammond, Xiao, Boone, *et al*. 2011). A single pass of the first 84 knockout plates has revealed 24 candidate MSUD genes, including *sad-3* to *sad-7* (Xiao *et al*. 2019). In this work, during the screening of knockout plates 85–87, we identified FGSC 18466 as a possible MSUD-deficient mutant. This deletion strain is missing the *ncu09093* gene, which has been replaced with the hygromycin resistance marker (*hph*). To verify that *ncu09093* actually plays a role in silencing, we subjected it to a quantitative MSUD suppression assay.

**Fig. 1.**
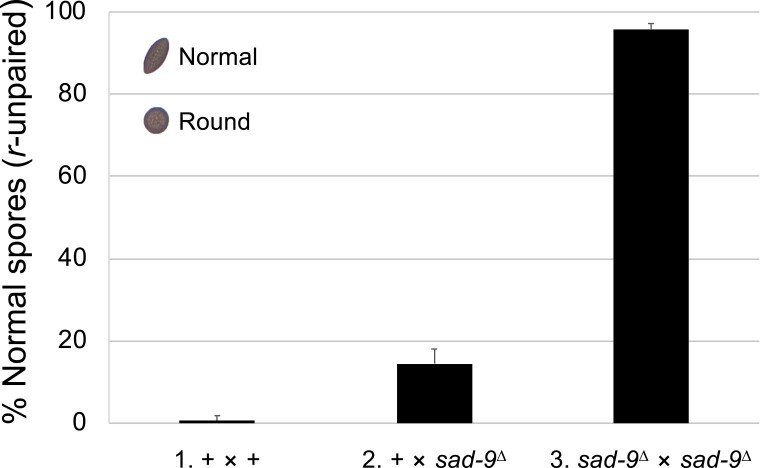
*sad-9* (*ncu09093*) is important for MSUD. In *Neurospora*, a cross typically produces American football-shaped ascospores. In each of the crosses examined here, one of the parents is missing the *round spore* (*r*^+^) gene. When MSUD is proficient, the unpaired *r*^+^ gene is silenced, leading to the production of mostly round progeny (0.67% football; cross 1). However, if an *r*-unpaired cross is heterozygous for *sad-9*^Δ^, the percentage of normal progeny increases considerably (14.5% football; cross 2), suggesting that the silencing mechanism is impaired. When both parents contain the *sad-9*^Δ^ mutation, nearly all the progeny are of normal shape (95.7% football; cross 3). +, wild type at pertinent loci. Crosses: (1) F2-29 × P3-08. (2) F2-29 × P23-09. (3) F9-03 × P22-49.

Normally, *Neurospora* produces American football-shaped ascospores. However, in a cross where only one *round spore* gene is present (i.e. *r*^+^ × *r*^Δ^), it is unpaired and subject to silencing (Shiu *et al*. 2001), resulting in mostly round progeny (or 0.67% football; Fig. 1, cross 1). This aberrant phenotype can be alleviated if silencing is deficient, for example, by having an unpaired MSUD gene in the cross (i.e*. sad*^+^ × *sad*^Δ^). This self-defeating mechanism is known as “silencing the silencer.” As shown in Fig. 1 (cross 2), when one parent of an *r*-unpaired cross contains the *ncu09093* deletion, 14.5% of the progeny are football-shaped. This suggests that *ncu09093*^Δ^ acts as a semidominant suppressor of MSUD. In accordance with our naming convention, *ncu09093* is hereafter referred to as *sad-9* (*suppressor of ascus dominance-9*).

### SAD-9 is a DEAD-box RNA helicase

*sad-9* is located on the left arm of linkage group I and encodes a 593-residue polypeptide. Based on a BLASTP search, SAD-9 has a region (residues 134–367) that can be classified as a “DEAD-box helicase domain of ATP-dependent RNA helicases DDX3 and DDX4” (conserved domain number: cd17967; *E*-value: 3.44e−91). The DDX3 (DEAD-box polypeptide 3) subfamily includes DDX3X (*Homo sapiens*), PL10 (*Mus musculus*), Belle (*D. melanogaster*), and Ded1 (*Saccharomyces cerevisiae*) (Sharma and Jankowsky 2014), whereas the DDX4 subfamily includes DDX4 (*H. sapiens*), Vasa (*D. melanogaster*), and GLH-1 (*Caenorhabditis elegans*) (Gustafson and Wessel 2010). Both *Drosophila* Belle and Vasa can partially complement a *DED1*-null mutant in yeast, indicating some functional conservation between the two closely related subfamilies (Senissar *et al*. 2014). A phylogenetic study suggests that the DDX4 subfamily originated from a duplication of an ancestral DDX3 gene early in metazoan evolution (Mochizuki *et al*. 2001; Gustafson and Wessel 2010), providing an explanation on why DDX4 proteins are ubiquitous among metazoans but absent in plants and fungi. Unsurprisingly, SAD-9 is more similar to DDX3 helicases than to DDX4 helicases (e.g. its BLASTP *E*-values with human DDX3X and DDX4 are 4e−119 and 1e−96, respectively).

### *sad-9* is expressed strongly in the sexual cycle

To ascertain the expression pattern of *sad-9*, we examined its transcript levels using RNA-seq datasets (see *Materials and methods*). Like other MSUD genes listed in Table 2, *sad-9* has a robust expression level in the sexual cycle (163 FPKM), consistent with its role in meiotic silencing. While *sad-9* has a relatively low vegetative expression (when compared with its sexual expression), it is still easily detectable (1.87 FPKM), leaving open the possibility that *sad-9* may have a function in the asexual cycle.

**Table 2.** Expression profiles of silencing genes.

Gene name	Gene no.	Vegetative expression (FPKM)	Sexual expression (FPKM)
Housekeeping			
* β-tubulin*	*ncu04054*	1222.0460	223.5137
MSUD			
* sad-2*	*ncu04294*	0.0000	38.5137
* sad-9*	*ncu09093*	1.8735	163.1941
*sms-2*	*ncu09434*	0.0496	673.0190
MSUD/Quelling			
* dcl-1*	*ncu08270*	4.4300	31.0978
* qip*	*ncu00076*	18.6841	107.2514

Quelling (vegetative silencing) and MSUD (sexual silencing) are two RNAi-based mechanisms in *Neurospora* (Gladyshev 2017). FPKM, fragments per kilobase of exon per million mapped reads.

### *sad-9* is crucial for sexual development and not vegetative growth

**Fig. 2.**
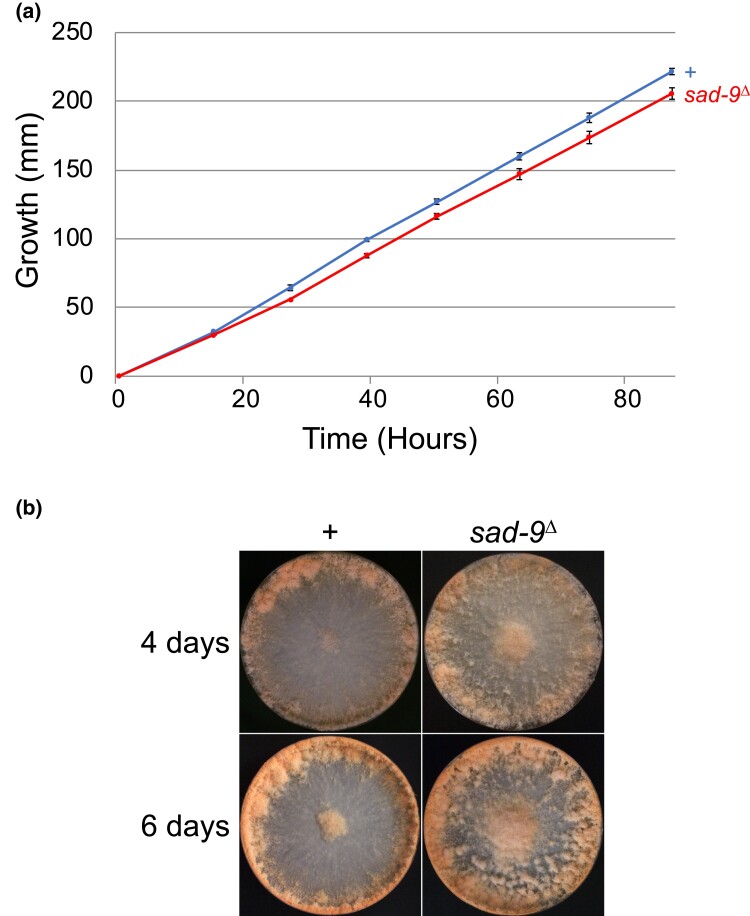
*sad-9* is not required for either vegetative growth or conidial development. a) Deletion of *sad-9* does not appear to have a substantial effect on the linear growth of the fungus. b) A *sad-9*^Δ^ strain is proficient in conidial production. Strains: P3-08 and P27-38.

**Fig. 3.**
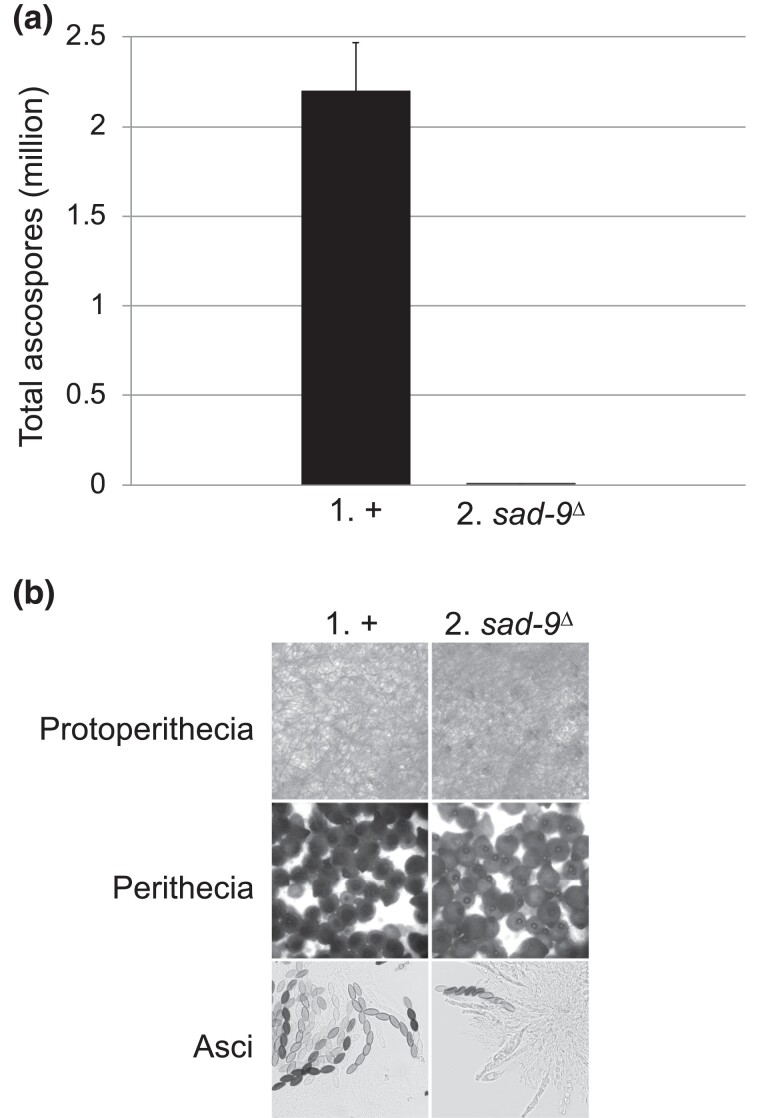
Deletion of *sad-9* leads to a severe reduction in sexual sporulation. a) A cross devoid of SAD-9 produces only a small fraction of the ascospores seen in a normal cross (1,770 *versus* 2.2 million). b) A cross homozygous for *sad-9*^Δ^ is proficient in protoperithecial and perithecial development. However, rampant ascus abortions can be observed upon dissection of the mutant perithecia. Crosses: (1) F2-01 × P3-08. (2) F9-03 × P23-09.

In *S. cerevisiae*, most DEAD-box RNA helicases are indispensable for cell growth (Linder and Fuller-Pace 2015). If *sad-9* were to be functional in the asexual cycle of *Neurospora*, it would likely be nonessential for cell viability and development, as its absence does not substantially affect linear growth and conidial production (Fig. 2). In contrast, *sad-9* is crucial for the sexual cycle. In a cross missing *sad-9*, the ascospore production drops more than a thousandfold (Fig. 3a). The fertility problem seems to lie in ascus development, as a *sad-9*-null cross has mostly aborted asci (Fig. 3b). Sexual defects have also been seen in other *sad*-null crosses (Shiu *et al*. 2001, 2006; Hammond, Xiao, Boone, *et al*. 2011; Samarajeewa *et al*. 2017).

### SAD-9 is an essential part of MSUD

For an MSUD gene that is absolutely required for ascospore production, it is not easy to evaluate its true significance to silencing, as we can only reduce its expression through the “silencing the silencer” knockdown. However, since a cross without *sad-9* can still produce some ascospores, we can determine its real impact on MSUD. As seen in Fig. 1 (cross 3), a cross devoid of *sad-9* yields predominantly unsilenced progeny, suggesting that this gene plays a critical (and not an auxiliary) role in the silencing pathway.

### SAD-9 localizes in the perinuclear region

**Fig. 4.**
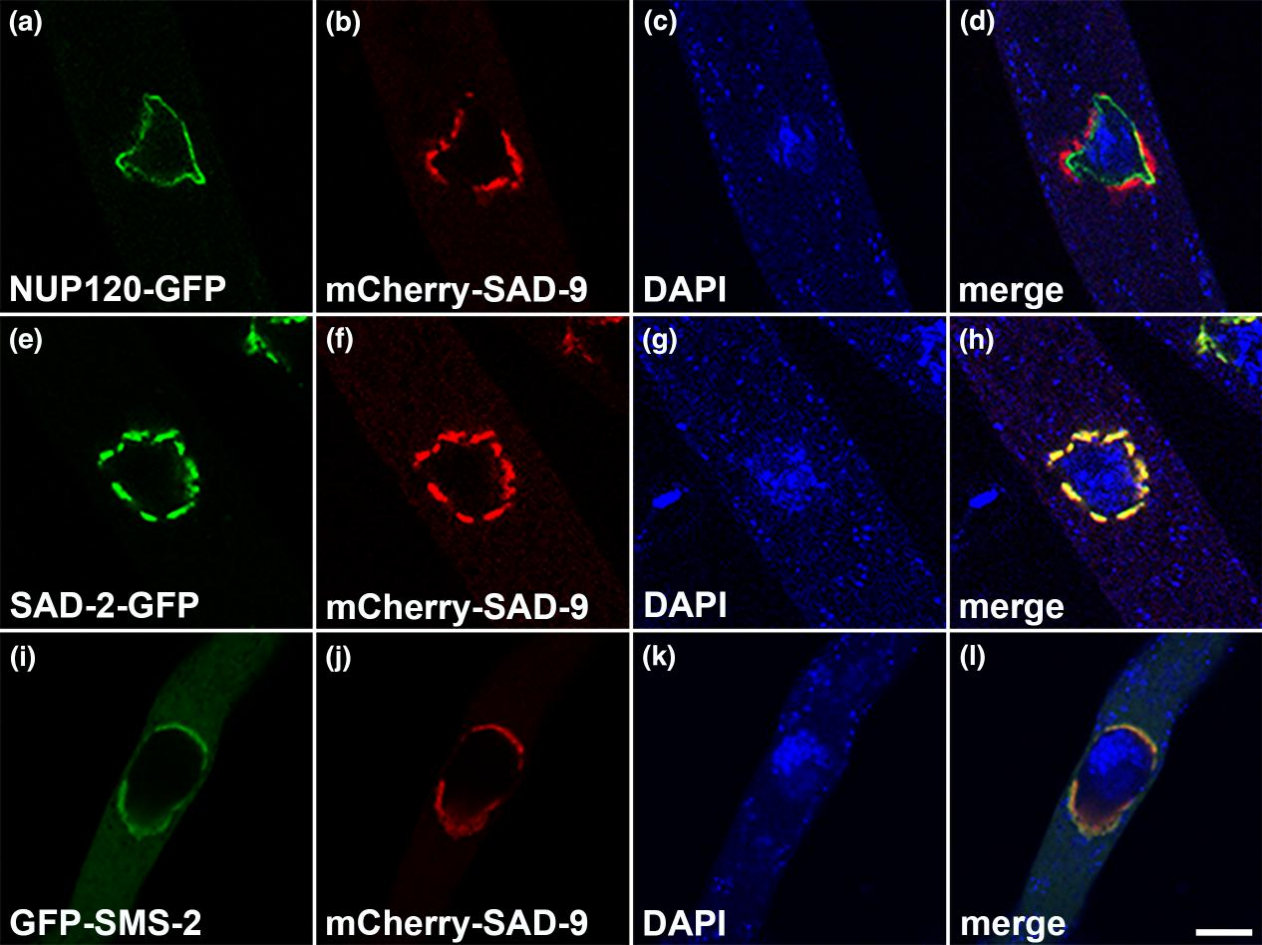
SAD-9 colocalizes with other silencing proteins. SAD-9 can be found in the perinuclear region (a–d), surrounding the nuclear envelope (as labeled by nucleoporin NUP120). SAD-9 colocalizes with the SAD-2 scaffold protein (e–h) and the SMS-2 Argonaute (i–l), two other MSUD proteins. Micrographs illustrate prophase asci expressing (a–d) *nup120-gfp* and *mCherry-sad-9* (P22-40 × P22-41), (e–h) *sad-2-gfp* and *mCherry-sad-9* (P22-38 × P22-39), and (i–l) *gfp-sms-2* and *mCherry-sad-9* (P22-47 × P22-48). The chromatin was stained with DAPI. Bar, 5 μm.

Many MSUD proteins localize in the perinuclear region, the center of silencing activity. Some of these proteins form the meiotic silencing complex, which seeks out and modifies certain RNA molecules (Decker *et al*. 2015). On the other hand, silencing proteins such as SAD-5 and SAD-6 are found in the nucleus (Hammond, Xiao, *et al*. 2013; Samarajeewa *et al*. 2014). To determine the subcellular localization of SAD-9, we tagged it with the mCherry fluorescent protein. As shown in Fig. 4d, SAD-9 resides in the perinuclear region, right outside of the nuclear envelope. As a matter of fact, SAD-9 colocalizes with SAD-2 and SMS-2, two other perinuclear MSUD proteins (Fig. 4, h and l).

### SAD-9 interacts with other silencing proteins

**Fig. 5.**
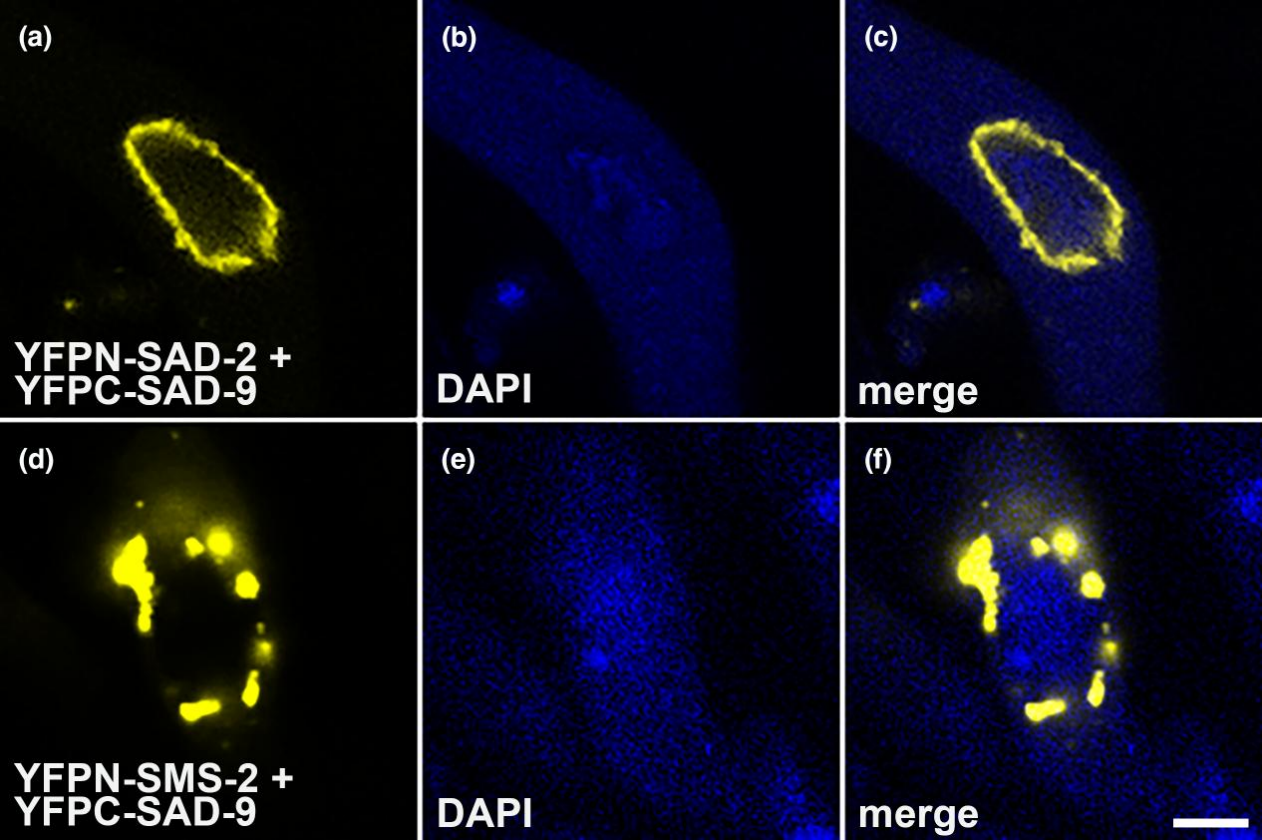
BiFC analysis. Here, the interaction between two proteins brings together the complete yellow fluorophore. SAD-9 interacts with both SAD-2 (a–c) and SMS-2 (d–f). Micrographs illustrate prophase asci expressing (a–c) *yfpn-sad-2* and *yfpc-sad-9* (P27-36 × P27-37) and (d–f) *yfpn-sms-2* and *yfpc-sad-9* (P27-32 × P27-33). The chromatin was stained with DAPI. Bar, 5 μm.

SAD-2 is a scaffold protein that recruits the SMS-2 Argonaute, among others, to the perinuclear region (Shiu *et al*. 2006; Decker *et al*. 2015). Since SAD-9 colocalizes with these two MSUD proteins, we asked whether it interacts with them. Using BiFC, we found that SAD-9 indeed has interaction with both SAD-2 and SMS-2, suggesting a direct connection among these components of the silencing pathway (Fig. 5).

### Sequential tethering of MSUD factors to the perinuclear region

**Fig. 6.**
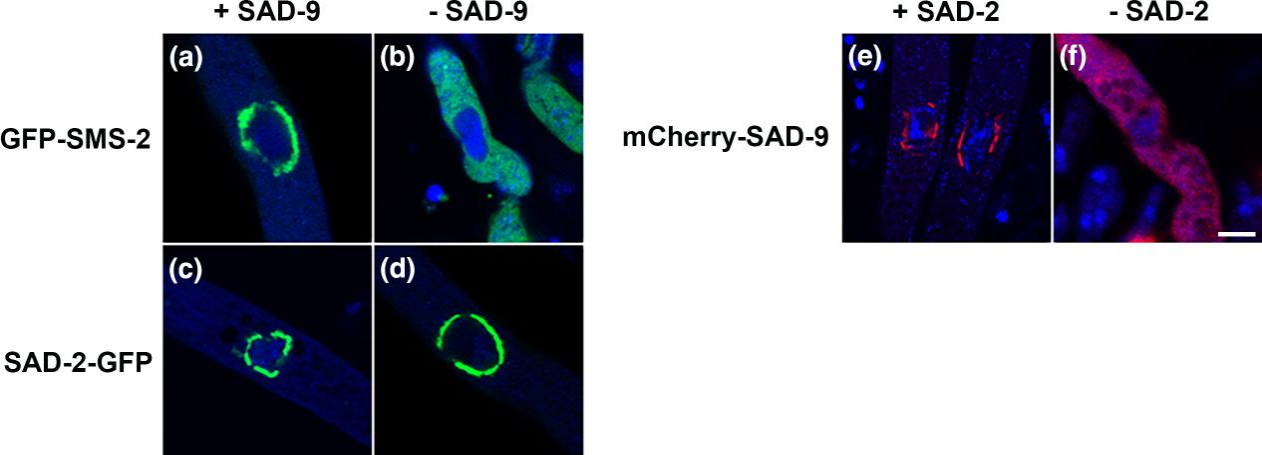
SAD-2 tethers SAD-9 and subsequently SMS-2 to the perinuclear region. When SAD-9 is present, SMS-2 localizes in the outer periphery of the nucleus (a). In the absence of SAD-9, SMS-2 is found in the bulk cytoplasm (b). SAD-2 is required for the perinuclear localization of SAD-9 (e and f), but not vice versa (c and d). Micrographs illustrate prophase asci from (a) F5-06 × P15-14, (b) P27-22 × P27-23, (c) P28-47 × P28-48, (d) P27-24 × P27-25, (e) P22-30 × P22-31, and (f) P22-36 × P22-37. The chromatin was stained with DAPI. Bar, 5 μm.

In *Bombyx mori*, Vasa acts as an assembly platform for Argonautes and others (Xiol *et al*. 2014). In *C. elegans*, GLH-1 promotes the localization of the PRG-1 Argonaute at perinuclear granules (Chen *et al*. 2020). Accordingly, we asked if SAD-9 recruits the SMS-2 Argonaute to the perinuclear region in *Neurospora*. In a *sad-9*-null cross, SMS-2 is diffused throughout the cytoplasm, suggesting that its perinuclear localization requires SAD-9 (Fig. 6, a and b).

Since both SAD-2 and SAD-9 control the placement of SMS-2, we wondered if the two SAD proteins play a role in each other's localization. As seen in Fig. 6 (c–f), while SAD-9 depends on SAD-2 for its perinuclear localization, the reverse is not true. All in all, our results are in line with the model that the SAD-2 scaffold recruits SAD-9, which subsequently recruits SMS-2, to the perinuclear region.

## Discussion

We have previously implicated CGH-1 and SAD-3, two RNA helicases, in meiotic silencing (Hammond, Xiao, Boone, *et al*. 2011; Xiao *et al*. 2021). Here, we have discovered yet another RNA helicase that is important for this surveillance mechanism. While some proteins only play a supplementary role in MSUD (e.g. the nuclear cap-binding complex; Decker *et al*. 2017), SAD-9 is an essential component of this pathway. This is evident from the observation that its elimination leads to a near-complete suppression of silencing.

Although some MSUD proteins are dispensable for the sexual cycle (Hammond, Xiao, *et al*. 2013; Samarajeewa *et al*. 2014; Boone *et al*. 2020), SAD-9 is vital to this stage. In animals, various DDX3 and DDX4 helicases have been associated with germline development (Lasko 2013). In *Neurospora*, the SMS-2 Argonaute is required for ascus formation (Wang *et al*. 2014). Since SAD-9 promotes the proper localization of SMS-2, it is not surprising that its absence has a devastating effect on ascospore production.

For its perinuclear localization, the worm GLH-1 helicase relies on its N-terminal FGG repeats (possibly through their interactions with the FG meshwork of nuclear pore proteins) and the MIP-1/2 scaffolds (Chen *et al*. 2020; Cipriani *et al*. 2021). Since SAD-9 lacks FGG repeats and the potential to be recruited by MIP-1/2 (which are not found in *Neurospora*), it must employ a different mechanism to anchor itself to the perinuclear region. Indeed, our results are consistent with the notion that the SAD-2 scaffold tethers SAD-9 to the outer nuclear periphery.

Besides recruiting the SMS-2 Argonaute to the perinuclear region, SAD-9 may also assist in its silencing process. For example, as an RNA helicase, SAD-9 could unwind secondary structures of a transcript and allow its sequence to be scanned by an siRNA-bound Argonaute. Alternatively, said Argonaute could utilize SAD-9's helicase activity to release itself from a processed mRNA target and move on to the next one (Dai *et al*. 2022). Future research in this direction will hopefully shed light on the mechanistic roles of SAD-9 and its associated factors.

## Supplementary Material

jkad083_Supplementary_Data

## Data Availability

Strains are available upon request. Supplemental material is available at G3 online.
