# Supplementary material for: A DEAD-box RNA helicase mediates meiotic silencing by unpaired DNA
Source: G3 (Bethesda). 2023 Apr 13;13(8):jkad083. doi: 10.1093/g3journal/jkad083 (PMC10411587; doi:10.1093/g3journal/jkad083)
Supplement: jkad083_Supplementary_Data [file jkad083_supplementary_data.pdf]

Table S1. Primers for strain construction and confirmation.

Purpose	Primer	Sequence (5′ to 3′)
gfp/yfpn-sms-2 construction	SMS-2-E	GTCCACTTGGTGCCATTCCCACT
	SMS-2-NGFP1	GCAGCCTGAATGGCGAATGGACGCGCGCGGAGGGTGTCAAAACTCACAA
	SMS-2-NGFP2	CAGGAGCGGGTGCGGGTGCTGGAGCGATGTCTGCTCCTGGCTCTCCC
	SMS-2-F	GTGCCATTCTGCTGCTTCCAGTT
	SMS-2-G	CACTTGCCTACCACGCCATGATT
	SMS-2-H	TGCTCAAACCGCCGTAATTGTTG
mCherry/yfpc-sad-9 construction	SAD-BP-E	GGCGGAGAGAAAGGGACAGATGG
	SAD-BP-NGFP1	GCAGCCTGAATGGCGAATGGACGCGCGCCGAAGAGGAGAGGTGACGAGA
	SAD-BP-NGFP2	CAGGAGCGGGTGCGGGTGCTGGAGCGATGGCTGACCACGGTTGGGGT
	SAD-BP-F	TGCTTGATGTTGGAGTGGGTGCT
	SAD-BP-G	GTCCGTATCTCCCAACCGCAATC
	SAD-BP-H	GCATCTCATCTGCCTCGTCAATCA
nup120-gfp construction	NCU02742-CT-LL	GTACGACTGTGCAACGGCATAGC
	NCU02742-CT-LR	CAGCGCCTGCACCAGCTCCTGCCCCCGACATGAGGATGTCCCCATCGTCACC
	NCU02742-CT-RL	CTCCTTCAATATCAGTTAACAAGGGCATGTTGGGCTGCTTGATTGTCCT
	NCU02742-CT-RR	CTTGGTGCGGTTTGAGGTTGGT
	NCU02742-CT-NL	AGACAGATAGGTGAAGGCGACAAGG
	NCU02742-CT-NR	AAGTGGAGCAAGCGAGCGAAAG
r$^{\Delta}$*::hph* confirmation	RSP-988505F	GCCACCTTTCCAACCCAATAATGC
	RSP-993102R	TGCCTTTTCAGCTGGAGACCAAGAC
sad-2$^{\Delta}$*::hph* confirmation	SAD-2-78812F	TTTGACATCCCGCTATCAATGCAG
	SAD-2-85682R	TGCTGTAGGAATGGCCATGGCTC
sad-2-gfp construction	SAD-2-A	GGGATTTATGCCCGCGTTCTCTA
	SAD-2-GFP1	CAGCGCCTGCACCAGCTCCTGCCCCATGTGGCTGCGTTCGAGCGTAGTGG
	SAD-2-GFP2	CTCCTTCAATATCAGTTAACAAGGGCAGGGGAAGGCAACAAACAATCCA
	SAD-2-B	CTTTCAGCAGCCCCCAACAACTC
	SAD-2-C	CGCCCATCATAACTTCCGAGTCAA
	SAD-2-D	GCCTTCTCCAGCCCATCACAACT
sad-9$^{\Delta}$*::hph* confirmation	NCU09093-F1	CCAGGGCGTTAGTGATGGCTTCT
	NCU09093-R1	TGCTCCTTCCTCGTCCCAGTCTT
yfpn-sad-2 construction	SAD-2-E	GATACCCGTGATGATTCTGATGA
	SAD-2-NGFP1	GCAGCCTGAATGGCGAATGGACGCGCGAACCGCCTGGACTGGATAGC
	SAD-2-NGFP2	CAGGAGCGGGTGCGGGTGCTGGAGCGATGACGGACCGCGACAAGAATGG
	SAD-2-F	CAGCACGAAGACGGGTGAGTGT
	SAD-2-G	TGTATCCGCAACCTGTAAAAGTCGTCA
	SAD-2-H	CCGGCGAAAGTGGAGAAGATTCA

Primers for DJ-PCR-based fluorescent tagging were designed as previously specified (Hammond *et al*. 2011b; Boone *et al*. 2020).
